# Higher Non-processed Red Meat Consumption Is Associated With a Reduced Risk of Central Nervous System Demyelination

**DOI:** 10.3389/fneur.2019.00125

**Published:** 2019-02-19

**Authors:** Lucinda J. Black, Gabrielle S. Bowe, Gavin Pereira, Robyn M. Lucas, Keith Dear, Ingrid van der Mei, Jill L. Sherriff, the Ausimmune Investigator Group, Caron Chapman

**Affiliations:** ^1^School of Public Health, Curtin University, Perth, WA, Australia; ^2^Telethon Kids Institute, University of Western Australia, Perth, WA, Australia; ^3^Research School of Population Health, National Centre for Epidemiology and Population Health, The Australian National University, Canberra, ACT, Australia; ^4^Centre for Ophthalmology and Visual Science, University of Western Australia, Perth, WA, Australia; ^5^School of Public Health, University of Adelaide, Adelaide, SA, Australia; ^6^Menzies Institute for Medical Research, University of Tasmania, Hobart, TAS, Australia

**Keywords:** red meat, processed meat, Ausimmune Study, multiple sclerosis, diet

## Abstract

The evidence associating red meat consumption and risk of multiple sclerosis is inconclusive. We tested associations between red meat consumption and risk of a first clinical diagnosis of central nervous system demyelination (FCD), often presaging a diagnosis of multiple sclerosis. We used food frequency questionnaire data from the 2003–2006 Ausimmune Study, an incident, matched, case-control study examining environmental risk factors for FCD. We calculated non-processed and processed red meat density (g/1,000 kcal/day). Conditional logistic regression models (with participants matched on age, sex, and study region) were used to estimate odds ratios (ORs), 95% confidence intervals (95% CI) and *p-*values for associations between non-processed (*n* = 689, 250 cases, 439 controls) and processed (*n* = 683, 248 cases, 435 controls) red meat density and risk of FCD. Models were adjusted for history of infectious mononucleosis, serum 25-hydroxyvitamin D concentrations, smoking, race, education, body mass index and dietary misreporting. A one standard deviation increase in non-processed red meat density (22 g/1,000 kcal/day) was associated with a 19% reduced risk of FCD (AOR = 0.81; 95%CI 0.68, 0.97; *p* = 0.02). When stratified by sex, higher non-processed red meat density (per 22 g/1,000 kcal/day) was associated with a 26% reduced risk of FCD in females (*n* = 519; AOR = 0.74; 95%CI 0.60, 0.92; *p* = 0.01). There was no statistically significant association between non-processed red meat density and risk of FCD in males (*n* = 170). We found no statistically significant association between processed red meat density and risk of FCD. Further investigation is warranted to understand the important components of a diet that includes non-processed red meat for lower FCD risk.

## Introduction

Diet is potentially a modifiable risk factor for multiple sclerosis (MS); however, the current evidence in inconclusive. The literature is somewhat consistent for higher intake of fish and very long-chain omega-3 polyunsaturated fatty acids (VLCn3PUFA: eicosapentaenoic acid, docosapentaenoic acid and docosahexaenoic acid) and reduced risk of MS (1–4) Studies investigating red meat consumption and risk of MS have returned inconclusive results (4–10).

Non-processed red meat is an important dietary source of protein, iron, zinc, vitamin B12, other minerals and vitamins, and VLCn3PUFA (11). However, epidemiological studies have linked higher red meat consumption with an increased risk of various chronic diseases, including obesity, type 2 diabetes, cardiovascular diseases and cancers. The findings have been attributed largely to the fat content of red meat, and the possible formation of carcinogenic compounds by cooking at high temperatures (12). It has also been postulated that long-term ingestion of N-glycolylneuraminic acid from consumption of red meat may promote inflammation and cancer progression (13), and may be a risk factor for MS (14). The strongest evidence around red meat consumption and chronic diseases is for higher processed meat intake and increased risk of colorectal cancer (15) and all-cause mortality (16); similar associations with other cancers have also been observed (17).

The 2003–2006 Australian Multi-center Study of Environment and Immune Function (Ausimmune Study) was a multi-center, case-control study examining associations between environmental factors and risk of a first clinical diagnosis of CNS demyelination (FCD) (18). Given that many people change their diet when diagnosed with MS (19, 20), associating dietary factors with risk of FCD offers the best opportunity to assess dietary risk factors before disease-related changes in behavior occur. We recently showed that a higher healthy dietary pattern score (high in poultry, fish, eggs, vegetables, and legumes) was associated with reduced risk of FCD in the Ausimmune Study (21). Also in the Ausimmune Study, there was no evidence that saturated or total fat intake in the preceding 12 months was associated with an altered risk of FCD (2); however, fat intake was from all dietary sources, and meat consumption was not specifically investigated. To better understand the relationship between meat consumption and risk of MS, we examined associations between red meat consumption (non-processed and processed) and risk of FCD in the Ausimmune Study.

## Methods

### Study Design

The Ausimmune Study was conducted between November 2003 and December 2006 in four regions of Australia: Brisbane city (latitude 27°S), Newcastle region (33°S), Geelong and the Western districts of Victoria (37°S), and the island of Tasmania (43°S) (18). Participants aged between 18 and 59 years and presenting with a FCD were notified to the study by a range of clinicians including neurologists, ophthalmologists, general physicians and, occasionally, general practitioners. Case eligibility was reviewed by a study neurologist after notification to the study (18). A study neurologist conducted a full history and neurological examination, confirmed the date of onset, and confirmed that the presenting symptoms indicated CNS demyelination (18). A total of 282 case participants were recruited, with a participation rate of 91% (22). Case participants had had an incident FCD within the study period, among the following types: a classic first demyelinating event (FDE; defined as a single, first, episode of clinical symptoms suggestive of CNS demyelination; *n* = 216); a first recognized event, but past history revealed a prior event highly suggestive of CNS demyelination (*n* = 48); first presentation of primary progressive MS [based on neurological assessment on study entry (*n* = 18)]. Although some participants may have had a previous undiagnosed demyelinating event, it is unlikely that a previous, unrecognized, demyelinating event would have triggered changes in dietary behavior.

We used the date of the MRI scan, which preceded the diagnosis of FCD by only a few days and was available for most case participants, as the date of diagnosis. Due to patient and health care factors, there was a time lag from the date of MRI scan to study interview. For participants with dietary intake data, the median [interquartile range (IQR)] time lag from the date of MRI scan by the neurologist (the date of the diagnosis which brought the participants into the study) to the study interview was 101 (147) days.

Control participants (*n* = 558) were randomly selected from the general population via the Australian Electoral Roll (compulsory registration for citizens ≥18 years). The control participation rate was 60% of those contacted (22). Control participants were matched to case participants based on age (within 2 years), sex and study region. To maximize the study power, between one and four matched controls were matched to each case, with more controls per case in regions with a lower expected number of cases. However, for practical reasons, these ratios were altered during the course of the study so that all centers were recruiting two controls per case in 2006.

The study was conducted in accordance with the Declaration of Helsinki. Ethics approval was obtained from the nine Human Research Ethics Committees of the participating institutions, namely: Barwon Health Research and Ethics Advisory Committee (ref 03/46); Ballarat Health Services and St John of God Health Services Committee; Royal Brisbane & Women's Hospital & Health Service District Office of the Human Research Ethics Committee (ref 2003/093); The University of Queensland Medical Research Ethics Committee (ref 2003000253); The Princess Alexandra Hospital Human Research Ethics Committee (ref 2004/059); The Queensland institute of Medical Research Human Research Ethics Committee [ref H0511-061 (P950)]; Hunter Area Research Ethics Committee (ref 03/06/11/3.07); Southern Tasmania Heath and Medical Research Ethics Committee (ref H7436); and The Australian National University Human Research Ethics Committee (ref 2002/111). All participants gave written informed consent for the use of their data. All participant information was anonymized and de-identified prior to analysis.

### Dietary Assessment

We used the Cancer Council Victoria Dietary Questionnaire for Epidemiological Studies version 2 (DQESv2) to collect information on habitual dietary intakes in the 12 months prior to the study interview (23). The DQES is a self-administered, semi-quantitative, food frequency questionnaire (FFQ) designed for use in the ethnically-diverse adult Australian population. The development of the DQES has been outlined in detail elsewhere (24). In brief, the food list on the original FFQ was based on weighed food records from 810 male and female volunteers, and further developed in a sample of 17,949 healthy men and women aged 40–69 years living in Melbourne, Australia (23).

The FFQ was validated relative to seven-day weighed food records in 63 women of child-bearing age for an iron supplementation study, where it performed as well as other validated FFQs: mean intakes from the weighted food record and the DQES were within ±20% for 21 of 27 nutrients (25). The FFQ is not recommended for children or younger people in general (23), but is considered a suitable tool for assessing intakes of foods and nutrients among adults in epidemiological studies, subject to the limitations common to all FFQs (23).

The DQESv2 measured consumption of food items from four groups: (1) cereals, sweets, and snacks; (2) dairy, meats, and fish; (3) fruit; (4) vegetables. The frequencies of consumption of food items were recorded according to the following scale: never, less than once per month, one to three times per month, once per week, twice per week, three to four times per week, five to six times per week, once per day, twice per day, and three or more times per day. Portion size diagrams of four commonly consumed foods (potato, other vegetables, steak, casserole), were used to determine respondents' average portion size factor as below median, median or above median (23). These portion size factors were then used to scale standard portion sizes up or down for different foods. Consumption of different types of alcoholic drink was recorded, including the total number of glasses usually drunk per day, and the maximum number of glasses drunk in any 24 h during the past year. Consumption of each of 101 food and beverage items was reported as grams per day. Non-processed red meat items reported from the FFQ were beef, pork, veal and lamb, and processed red meat items were ham, bacon, salami and sausage. Nutrient intakes for the DQESv2 were computed primarily using composition data from the Australian NUTTAB 95 database (26), with data for VLCn3PUFA derived from the Royal Melbourne Institute of Technology fatty acid database of Australian foods (27). Total energy intake (kcal/day) was calculated from food and beverage intakes combined.

### Covariates

Self-report questionnaires were used to collect information on the following characteristics: race (Caucasian, other); history of infectious mononucleosis (yes, no, don't know); total number of years smoking minus any periods of abstinence; and highest education (year 10 or less, year 12 and Technical and Further Education, university). Most participants (94%) provided a blood sample; serum aliquots (1 mL) were stored at −80°C and analyzed at study completion for 25-hydroxyvitamin D (25(OH)D) concentration (nmol/L) using liquid chromatography tandem mass spectrometry (18). Serum 25(OH)D concentrations for case participants were de-seasonalized using the seasonal pattern of control serum 25(OH)D concentrations, to account for blood samples of cases and controls being taken at different times of the year (18).

The study nurse measured height and weight, and body mass index (BMI) was calculated as weight in kilograms divided by height in meters squared. Basal metabolic rate was calculated using the equations developed by Harris and Benedict (28): males *h* = 66.4730+13.7516W+5.0033S−6.7750A; females *h* = 665.0955+9.5634W+1.8496S−4.6756A (where, *h* = kcal/day; W = weight in kilograms; S = stature in centimeters; A = age in years). Under-reporters were classified using the Goldberg cut-off point of below BMRx1.05 (29). A two-category variable was created for dietary misreporting: under-reporter and plausible reporter.

### Statistical Analysis

Of the 840 participants (282 cases, 558 controls) in the Ausimmune Study, 791 participants provided dietary intake data. We calculated non-processed (beef, veal, lamb, pork) and processed (ham, bacon, salami, sausages) red meat density as grams of meat intake per 1,000 kcal of energy intake per day (g/1,000 kcal/day). Statistical outliers in non-processed and processed red meat density were identified as those with values as follows: 75th percentile + [3 × interquartile range (IQR)] and 25th percentile-(3 × IQR). Three participants (two cases, one control) had non-processed red meat density values above the threshold of 132 g/1,000 kcal/day, and 10 participants (three cases, 10 controls) had processed red meat density values above the threshold of 50 g/1,000 kcal/day. These participants were excluded from further analyses. Participants with missing data for covariates were also excluded from further analyses. After removing unmatched cases and controls, data for a total of 689 participants (250 cases, 439 controls) were available for non-processed red meat analyses, and for 683 participants (248 cases, 435 controls) for processed red meat analyses.

Characteristics of case and control participants in the current study were described as frequencies and percentages for categorical variables, mean and standard deviation (SD) for continuous variables with distributions resembling a Normal distribution, and median and interquartile range (IQR) for continuous variables with a non-Normal distribution. Characteristics of control participants who were included in the final model (*n* = 439 for non-processed red meat; *n* = 435 for processed red meat) were compared with those who were excluded from the final model due to missing data or being unmatched to a case participant (*n* = 119 for non-processed red meat; *n* = 123 for processed red meat). Pearson's chi-square tests were used for categorical variables, independent samples *t*-tests for continuous variables with Normal distributions, and Mann-Whitney *U* tests for continuous variables with non-Normal distributions.

We used conditional logistic regression models (with cases and controls matched on age, sex and study region) to estimate odds ratios (ORs), 95% confidence intervals (95% CI) and *p-*values for associations between red meat density (non-processed and processed) and risk of FCD. We used non-processed and processed red meat density as the continuous variable [per standard deviation (SD)], with models conducted unadjusted and adjusted for known environmental risk factors for MS (history of infectious mononucleosis, serum 25(OH)D concentrations, total years of smoking), potential risk factors for MS (race, BMI, education) and dietary misreporting. We tested for non-linearity using a quadratic term for non-processed and processed red meat density. Models were run in the total sample and also stratified by sex. We investigated possible interactions between sex, red meat density (non-processed and processed) and risk of FCD using a multiplicative term. We tested whether any observed relationship between non-processed red meat consumption and risk of FCD was mediated by intakes of VLCn3PUFA by further adjusting models for VLCn3PUFA density (g/1,000 kcal/day).

We conducted the following sensitivity analyses in the total study sample and stratified by sex: (a) risk of FCD including only participants with plausible energy intakes (>500 to < 5,000 kcal/day); (b) risk of FCD in case participants who completed the study interview within 90 days from the date of MRI scan (in order to reduce the likelihood that participants had already changed their diet in response to a FCD, noting that the FFQ recorded data for the 12 months prior to the episode that brought the case participant into the study); and (c) within the smaller group of those with a classic FDE. Data were analyzed using Stata 14 software (30).

## Results

### Participant Characteristics

As expected, cases were more likely than controls to have a history of infectious mononucleosis, lower serum 25(OH)D concentrations and more years of smoking (Table 1). Median non-processed red meat density was lower in cases than controls, while there was no difference in median processed red meat density between cases and controls (Table 1). Median non-processed and processed red meat intake and density were higher in males than females (Table 2).

**Table 1:** Characteristics of participants in the Ausimmune Study included in the analysis for non-processed red meat (*n* = 689; 250 cases, 439 controls) and processed red meat (*n* = 683; 248 cases, 435 controls).

	**Non-processed red meat**		**Processed red meat**	
	**Case**	**Control**	**Case**	**Control**
**SEX, % (*****n*****)**^**1**^				
Male	25.2 (63)	24.4 (107)	25.4 (63)	24.1 (105)
Female	74.8 (187)	75.6 (332)	74.6 (185)	75.9 (330)
Age, year, mean (SD)^a^	38.7 (9.7)	40.0 (9.6)	38.8 (9.7)	40.0 (9.6)
**STUDY REGION, % (*****n*****)**^**a**^				
Brisbane (27°S)	34.0 (85)	37.1 (163)	33.9 (84)	37.2 (162)
Newcastle (33°S)	12.4 (31)	14.6 (64)	11.7 (29)	14.0 (61)
Geelong (37°S)	23.6 (59)	24.4 (107)	24.2 (60)	24.6 (107)
Tasmania (43°S)	30.0 (75)	23.9 (105)	30.2 (75)	24.1 (105)
Race, % (n)	94.1 (241)	94.1 (413)	96.4 (239)	94.0 (409)
Caucasian	3.6 (9)	5.9 (26)	3.6 (9)	6.0 (26)
**OTHER**				
**History of infectious mononucleosis, % (*****n*****)**				
No	65.2 (163)	79.3 (348)	64.9 (161)	79.3 (345)
Yes	28.0 (70)	16.2 (71)	28.2 (*70*)	16.1 (70)
Don't know	6.8 (17)	4.6 (20)	6.9 (17)	4.6 (20)
Serum 25(OH)D concentrations (nmol/L), mean (SD)	75.8 (29.7)	81.9 (30.3)	75.8 (29.8)	81.5 (30.6)
Total years of smoking, median (IQR)	5.7 (18.7)	2.0 (14.8)	5.4 (18.9)	1.7 (14.7)
**BODY MASS INDEX, MEDIAN (IQR)**				
**Education, % (*****n*****)**				
Year 10 or less	24.8 (62)	33.3 (146)	25.0 (62)	33.3 (145)
Year 12 and tertiary and further education	50.0 (125)	41.7 (183)	49.2 (122)	41.2 (179)
University	25.2 (63)	25.1 (110)	25.8 (64)	25.5 (111)
Non-processed red meat intake (g/day), median (IQR)	46.5 (62.1)	58.7 (57.8)	N/A	N/A
Non-processed red meat density (g/1,000 kcal/day), median (IQR)	28.9 (28.2)	35.4 (27.4)	N/A	N/A
Processed red meat intake (g/day), median (IQR)	N/A	N/A	18.9 (22.6)	18.3 (22.1)
Processed red meat density (g/1,000 kcal/day), median (IQR)	N/A	N/A	11.1 (11.2)	11.0 (10.5)
**DIETARY MISREPORTING, % (*****n*****)**				
Under-reporter	42.8 (107)	40.6 (178)	42.7 (106)	40.7 (177)
Plausible reporter	57.2 (143)	59.5 (261)	57.3 (142)	59.3 (258)

*SD, standard deviation; 25(OH)D, 25-hydroxyvitamin D; IQR, interquartile range*.

a *Case and control participants were matched on sex, age (within 2 years) and study region*.

**Table 2:** Red meat intake (g/day) and density (g/1,000 kcal/day) for male and female participants in the Ausimmune Study included in the current study.

	**Males**				**Females**			
	***n***	**Case**	***n***	**Control**	***n***	**Case**	***n***	**Control**
**NON-PROCESSED RED MEAT**								
Intake (g/day), median (IQR)	63	87.4 (108.7)	107	79.4 (83.3)	187	39.1 (49.7)	332	54.2 (53.1)
Density (g/1,000 kcal/day), median (IQR)	63	38.6 (31.5)	107	36.5 (26.7)	187	26.9 (24.4)	332	35.05 (27.8)
**PROCESSED RED MEAT**								
Intake (g/day), median (IQR)	63	29.4 (33.7)	105	27.3 (34.3)	185	15.9 (20.0)	330	16.9 (19.6)
Density (g/1,000 kcal/day), median (IQR)	63	12.6 (11.4)	105	12.0 (10.3)	185	10.3 (10.6)	330	10.4 (10.6)

There was no difference between the control participants who were included in the main model for non-processed red meat density (*n* = 439) and those who were excluded from the main model (*n* = 119) with respect to the following characteristics: history of infectious mononucleosis (*p* = 0.85), serum 25-hydroxyvitamin D concentration (*p* = 0.14), total years of smoking (*p* = 0.87), race (*p* = 0.30), age (*p* = 0.52), education (*p* = 0.20), and BMI (*p* = 0.77). Compared with those excluded from the main model of non-processed red meat density, control participants included in the main model were more likely to be male (*p* = 0.001) and to be from Brisbane or Tasmania (*p* < 0.001). For processed red meat, there was no difference between the control participants who were included in the main model (*n* = 435) and those who were excluded from the main model (*n* = 123) with respect to the following characteristics: history of infectious mononucleosis (*p* = 0.80), total years of smoking (*p* = 0.59), race (*p* = 0.26), age (*p* = 0.62), education (*p* = 0.22), and BMI (*p* = 0.39). Compared with those excluded from the main model for processed red meat density, control participants included in the main model were more likely to be male (*p* = 0.02), to be from Brisbane or Tasmania (*p* < 0.001), and to have lower serum 25-hydroxyvitamin D concentrations (*p* = 0.04).

### Non-processed Red Meat Density and Risk of FCD

The test of a quadratic term for non-processed red meat density indicated no evidence of non-linearity (*p* = 0.51). In the total population, a one SD increase in non-processed red meat density (22 g/1,000 kcal/day) was associated with a 19% reduced risk of FCD in both unadjusted and adjusted models (Table 3). The effect was strongest in females, with a 27% and 26% reduced risk of FCD in unadjusted and adjusted models, respectively. There was no evidence of an association between non-processed red meat density and risk of FCD in males only. The interaction between sex and non-processed red meat density was borderline not statistically significant (*p* = 0.06). When further adjusted for VLCn3PUFA density, there was little change in the estimated effect of non-processed red meat density on risk of FCD (total population, AOR = 0.81; 95%CI 0.68, 0.96; *p* = 0.02; males, AOR = 1.08; 95%CI 0.72, 1.61; *p* = 0.71; females, AOR = 0.76; 95%CI 0.61, 0.94; *p* = 0.01).

**Table 3:** Results from conditional logistic regressions models showing associations between red meat density (non-processed and processed) and risk of FCD in participants of the Ausimmune Study included in the current study, in the total study sample and stratified by sex.

		**Unadjusted**		**Adjusted**^**a**^	
	***n* (cases, controls)**	**OR (95% CI)**	***P***	**AOR (95% CI)**	***P***
**NON-PROCESSED RED MEAT DENSITY (g/1,000 kcal/day, per SD)**					
Total	689 (250, 439)	0.81 (0.68, 0.95)	0.01	0.81 (0.68, 0.97)	0.02
Males	170 (63, 107)	1.06 (0.78, 1.45)	0.71	1.08 (0.73, 1.59)	0.69
Females	519 (187, 332)	0.73 (0.59, 0.89)	0.002	0.74 (0.60, 0.92)	0.01
**PROCESSED RED MEAT DENSITY (g/1,000 kcal/day, per SD)**					
Total	683 (248, 435)	1.00 (0.85, 1.17)	0.98	1.04 (0.88, 1.22)	0.67
Males	168 (63, 105)	0.92 (0.68, 1.24)	0.59	0.92 (0.66, 1.29)	0.62
Females	515 (185, 330)	1.07 (0.87, 1.30)	0.53	1.10 (0.90, 1.35)	0.36

*FCD, first clinical diagnosis of central nervous system demyelination; SD, standard deviation*.

a *Adjusted for history of infectious mononucleosis, serum 25-hydroxyvitamin D concentrations, total years of smoking, body mass index, race, education, and dietary misreporting*.

Findings were similar to the main models when limited to those with energy intakes within a plausible range (>500 to < 5,000 kcal/day) (Table 4). The findings were also similar, but with wider confidence intervals (as expected due to the smaller sample size), in analyses limited to case participants who completed the study interview within 90 days from the date of MRI scan, and in those with a classic FDE (Table 4).

**Table 4:** Results from conditional logistic regressions models showing associations between red meat density (non-processed and processed) and (a) risk of FCD including only participants with plausible energy intakes (>500 to < 5,000 kcal/day), (b) risk of FCD in case participants who completed the study interview within 90 days from the date of MRI scan, (c) risk of FDE.

		**Unadjusted**		**Adjusted**^**a**^	
	***n* (cases, controls)**	**OR (95% CI)**	***P***	**AOR (95% CI)**	***P***
**NON-PROCESSED RED MEAT DENSITY (g/1,000 kcal/day, per SD)**					
**(a) risk of FCD including only participants with plausible energy intakes (>500– < 5,000 kcal/day)**					
Total	676 (430, 246)	0.81 (0.69, 0.96)	0.02	0.82 (0.69, 0.98)	0.03
Males	161 (101, 60)	1.10 (0.80, 1.50)	0.57	1.14 (0.77, 1.70)	0.51
Females	515 (329, 186)	0.73 (0.59, 0.89)	0.002	0.74 (0.60, 0.92)	0.01
**(b) risk of FCD in case participants who completed the study interview within 90 days from the date of MRI scan**					
Total	316 (115, 201)	0.86 (0.68, 1.08)	0.20	0.87 (0.67, 1.11)	0.26
Males	93 (34, 59)	1.28 (0.82, 2.01)	0.28	1.69 (0.90, 3.16)	0.10
Females	223 (81, 142)	0.71 (0.53, 0.96)	0.03	0.71 (0.52, 0.97)	0.03
**(c) Risk of FDE**					
Total	519 (191, 328)	0.82 (0.68, 0.99)	0.04	0.80 (0.65, 0.98)	0.03
Males	129 (48, 81)	1.07 (0.75, 1.51)	0.72	1.07 (0.69, 1.66)	0.76
Females	390 (143, 247)	0.73 (0.58, 0.92)	0.01	0.71 (0.55, 0.92)	0.01
**PROCESSED RED MEAT DENSITY (g/1,000 kcal/day, per SD)**					
**(a) risk of FCD including only participants with plausible energy intakes (>500– < 5,000 kcal/day)**					
Total	670 (426, 244)	1.00 (0.85, 1.18)	0.98	1.04 (0.88, 1.23)	0.64
Males	159 (99, 60)	0.93 (0.68, 1.27)	0.64	0.92 (0.64, 1.32)	0.65
Females	511 (327, 184)	1.03 (0.86, 1.24)	0.75	1.08 (0.89, 1.31)	0.45
**(b) Risk of FCD in case participants who completed the study interview within 90 days from the date of MRI scan**					
Total	313 (114, 199)	1.00 (0.80, 1.25)	0.99	1.05 (0.82, 1.33)	0.70
Males	92 (34, 58)	1.00 (0.70, 1.43)	0.996	1.02 (0.64, 1.62)	0.93
Females	221 (80, 141)	1.00 (0.75, 1.32)	0.99	1.03 (0.76, 1.40)	0.83
**(c) Risk of FDE**					
Total	518 (190, 328)	0.99 (0.83, 1.18)	0.89	1.02 (0.85, 1.23)	0.81
Males	128 (48, 80)	0.90 (0.66, 1.24)	0.53	0.94 (0.65, 1.35)	0.75
Females	390 (142, 248)	1.03 (0.83, 1.27)	0.80	1.06 (0.85, 1.33)	0.58

*FCD, first clinical diagnosis of central nervous system demyelination; MRI, magnetic resonance imaging; FDE, incident classic first demyelinating event*.

a *Adjusted for history of infectious mononucleosis, serum 25-hydroxyvitamin D concentrations, total years of smoking, body mass index, race, education, and dietary misreporting*.

### Processed Red Meat Density and Risk of FCD

The test of a quadratic term for processed red meat density indicated no evidence of non-linearity (*p* = 0.90). There was no statistically significant association between processed red meat density and risk of FCD in the total population, nor when stratified by sex (Table 3). There was no statistically significant interaction between sex and processed red meat density (*p* = 0.56). Sensitivity analyses showed similar findings to the main models (Table 4).

## Discussion

Higher consumption of non-processed red meat was associated with a reduced risk of FCD: a reduction of 19% per one SD increase in non-processed red meat density. We found no evidence of an association between processed red meat consumption and risk of FCD. The findings were similar when limited to those who completed the study interview within 90 days of their MRI scan, which reduces the likelihood that participants were reporting on changes in their dietary behavior in response to their diagnosis.

Red meat contains both VLCn3PUFA and vitamin D, low levels of which have been associated with the risk and/or progression of MS (2, 22, 31, 32). VLCn3PUFA play critical roles in the central nervous system, exhibiting anti-inflammatory and neuro-protective effects (33). Indeed, we previously found that higher intake of VLCn3PUFA was associated with reduced risk of FCD in the Ausimmune Study (2). Australian beef is commonly from grass-fed livestock and has higher levels of VLCn3PUFA compared to grain-fed beef (34); beef and lamb have previously been shown to contribute 28% of the total VLCn3PUFA intake in Australia (35). However, adjusting for VLCn3PUFA density did not influence the effect estimate for non-processed red meat density, suggesting an association between non-processed red meat consumption and risk of FCD that is independent of VLCn3PUFA.

Red meat also contains vitamin D and its hydroxylated form, 25(OH)D (36). The latter is considered to have five times the bioactivity of vitamin D itself (37). Indeed, plasma 25(OH)D concentrations were higher in meat eaters than in vegetarians and vegans in a study of 2,107 British adults participating in the European Prospective Investigation into Cancer and Nutrition (EPIC)-Oxford cohort (38). Due to its frequency of consumption, red meat is likely to be the most significant source of dietary vitamin D in Australia (39). Since low vitamin D status is a known risk factor for MS (22), we adjusted for serum 25(OH)D concentrations, the major circulating form of vitamin D and the product of both sun exposure and dietary intake of vitamin D. Hence, our findings indicate an association between non-processed red meat consumption and risk of FCD that is independent of vitamin D.

When stratified by sex, the association between non-processed red meat and risk of FCD was observed only in females. The sample size for females was approximately 3 times greater than that for males, reflecting the higher prevalence of MS in females [approximately 2.5–3 times higher in females than males (40)]. Hence, the sex difference observed in our study may be a result of the substantially lower sample size for males. Alternatively, the lack of effect in males might be explained by the existence of a threshold intake of non-processed red meat consumption, above which risk is reduced. For males, the median non-processed red meat consumption for cases and their matched controls was 87 and 79 g/day, respectively, which was much greater than that for females (39 g/day for cases, and 54 g/day for controls). It is also plausible that we did not observe an association for males because of insufficient exposure contrast between the male cases and controls. The median difference in non-processed red meat consumption between male cases and their matched controls was 8 g/day. This was less than that for females (median difference = 15 g/day).

An alternative explanation for the observed sex difference relates to iron. From the onset of menarche until menopause, females have greater iron requirements than males (41), and nearly 40% of Australian females aged 14–50 years have inadequate iron intakes (42). Red meat is high in haem iron (43), which is more bioavailable than the plant form of iron (non-haem) (44). Hence, low red meat consumption may compromise iron status, and vegetarians (particularly female vegetarians) are at high risk of iron deficiency (45). Dysregulation of iron metabolism is implicated in MS, with degeneration of deep gray matter structures corresponding with iron accumulation (46). Conversely, iron levels may be reduced in the normal-appearing white matter, with the extent of iron depletion correlating with disease duration (47). There is no evidence of systematic iron overload in people with MS (48, 49). Therefore, it has been suggested that the iron dysregulation associated with MS is a redistribution of iron levels between different areas of the brain, rather than global iron accumulation (50). Iron sufficiency may be important in preventing MS since iron is involved in the synthesis, maintenance and repair of myelin, may be critical to oligodendrocyte activity and integrity, and plays an integral role in mitochondrial energy production (50). Indeed, a recent analysis of pediatric MS patients and controls from the United States (*n* = 312 cases; *n* = 456 controls) showed that low iron intake was associated with an increased risk of MS (51).

The previous literature on meat consumption and risk of MS is limited and mainly suggests an association between higher processed red meat consumption and increased risk of MS (Supplementary Table 1). In a 1992–1995 Canadian matched case-control study, intakes of approximately 40 foods and nutrients in the year prior to onset were estimated from a food frequency questionnaire (4). Approximately 15 foods and nutrients were significantly associated with increased risk of MS, including processed meat; there was no statistically significant association between beef consumption and risk of MS. A 1986–1991 matched case-control study in Croatia showed that weekly consumption (3–5 times/week) of a traditional meal of potatoes with lard and smoked meat was associated with increased risk of MS (5). This study was conducted in people with established MS, who had had MS for an average of 20 years, with only 9 participants showing no signs of disability, and food recall was from birth until the first signs of the disease appeared. Hence, our results are not comparable with that study.

A study by Lauer on 46 sociogeographic factors in the States of America showed a positive correlation between meat sales and incidence of MS (9); however, such ecological studies are low on the hierarchy of evidence. Another ecological study by Lauer using per capita meat consumption from 26 countries, showed that sausage preservation by smoking was associated with increased risk of MS (based on prevalence of MS from published sources) (6). The association was independent of meat and pork consumption (6). Lauer has suggested that the combination of curing meats with nitrites and subsequent smoking produces nitrophenylated compounds which conjugate with particular proteins in the CNS (7). Although our study, and an analysis of the Nurses' Health Study and Nurses' Health Study II (8), found no association between processed meat consumption and risk of FCD and MS, respectively, both studies assessed diet only in the year preceding diagnosis. Consumption of smoked meat during childhood and early adolescence may be more relevant to MS onset than current dietary habits (7, 10) and was not assessed in our study.

Our study has some important strengths compared with previous studies assessing meat consumption and risk of MS. We used a multi-center, matched case-control study design with each case participant reviewed by the study neurologist, who obtained a full clinical history and data from MRI scans. We collected dietary data soon after diagnosis of FCD, which reduced the likelihood that participants were reporting intakes based on disease-related changes in dietary behavior. To further reduce the possibility that the reported dietary intake was due to dietary changes adopted as a result of their diagnosis, we also conducted a sensitivity analysis in case participants interviewed within 90 days of their MRI scan; this was the shortest time interval that retained a reasonable sample size. Due to the comprehensive data collected in the Ausimmune Study, we were able to account for the known environmental risk factors for MS, including serum 25(OH)D concentrations measured by an internationally-standardized methodology (52). However, although we adjusted for known and potential risk factors for MS, we cannot rule out the possibility of residual confounding.

Although food frequency questionnaires are appropriate for dietary intake assessment in epidemiological studies due to their feasibility (both in terms of cost and participant burden), the under-reporting of energy intake by self-reported dietary methods is widely acknowledged (53). We, therefore, adjusted for dietary misreporting and conducted a sensitivity analysis including only participants with plausible energy intakes; this returned very similar results to the main model.

A limitation of our study was the assessment of diet only in the year prior to a FCD; it is possible that dietary habits in childhood or adolescence may influence later onset of MS. Furthermore, our results may not be generalizable to other populations: Australian beef is commonly from grass-fed livestock and is likely to have a different fat composition to beef from grain-fed livestock; and participants in the Ausimmune Study were predominantly Caucasian, and likely to follow dietary habits common to a Caucasian population living in Australia.

A limitation of all FFQs is that not all food items are captured. Consumption of game meats (e.g., kangaroo, goat, rabbit, and venison) was not captured in the DQES. However, it is unlikely that game meats were widely or frequently consumed; hence, unlikely that the omission of game meats from the FFQ would have influenced the results. Another limitation of the DQES is that it has been validated only in females aged 16–48 years, although it is widely used in epidemiological studies including both males and females.

Since the control participation rate was 60% of those contacted, the potential influence of selection bias must be considered. As is commonly reported in studies with less than full participation, the control sample was more highly educated than the general population (e.g., 31% of control participants in the Brisbane study region had completed university vs. 19% (54) of the general population in that region). Similarly, the case sample was more highly educated than the general population (e.g., 26% of case participants in the Brisbane study region had completed university vs. 19% (54) of the general population in that region). After adjusting for age, sex and study region, there was no statistically significant association between education level and non-processed red meat density (results not shown); hence, it is unlikely that any bias in education level would have affected our findings with respect to non-processed red meat. There was a statistically significant inverse association (*p* < 0.05) between education level and processed meat density (results not shown). However, had our control sample been more representative of the general population (i.e., with lower education level), the effect estimate would have been attenuated, with no likely effect on our null findings.

In summary, our results suggest a reduced risk of FCD with higher consumption of non-processed red meat. However, our study does not elucidate the important components of non-processed red meat that may be beneficial. It is important to note that in order to enhance dietary variety and reduce some of the health risks associated with consuming red meat, the maximum number of weekly serves of lean red meat recommended for Australian adults is seven (where one serve = 65 g) (55). In general, young Australian women are advised to eat more red meat, while Australian adult men need to eat less red meat (56). With respect to FCD, further investigation is warranted to understand the important components of a diet that includes non-processed red meat. It is also not known whether non-processed red meat consumption is associated with disease progression in people with established MS.

## Author Contributions

The Ausimmune Investigator Group designed the study. GB and LB analyzed the data. GP provided support with statistical analysis and interpretation of data. GB and LB wrote the paper. RL, JS, GP, KD, IvdM, and the Ausimmune Investigator Group provided critical revision of the manuscript for important intellectual content. LB had primary responsibility for the final content. All the authors read and approved the final version of the manuscript.

### Conflict of Interest Statement

The authors declare that the research was conducted in the absence of any commercial or financial relationships that could be construed as a potential conflict of interest.
