# Supplementary material for: Higher Non-processed Red Meat Consumption Is Associated With a Reduced Risk of Central Nervous System Demyelination
Source: Front Neurol. 2019 Feb 19;10:125. doi: 10.3389/fneur.2019.00125 (PMC6389668; doi:10.3389/fneur.2019.00125)
Supplement: Supplementary file 1 [file Data_Sheet_1.PDF]

Supplementary table 1. Previous studies assessing meat consumption and risk of MS

Reference	Study design	Study population	Predictor	Outcome	Results
Ghadirian et al 1998	Case-control	Canada (*n* =197 incident cases of MS; *n* =202 controls)	Food intakes from a food frequency questionnaire (g/day)	Risk of MS	Higher processed meat intake associated with increased risk of MS; no association between beef intake and risk of MS
Gusev et al 1996	Case-control	Moscow (*n* =155 people with MS; *n* =155 controls)	Predominant meat *vs.* vegetable diet during childhood	Risk of MS	A meat predominating diet in childhood was significantly associated with risk of MS
Lauer 1994	Ecological	USA	Annual sales of meat products in U.S. dollars in specialised food retail shops with a correction factor to account for total food sales	Risk of MS by State from published sources	Correlation between consumption of meat and MS rate
Lauer 2007	Ecological	26 countries	Per capita food consumption (total meat, pork, animal fat) and meat preservation methods (smoke *vs* . air-drying or equivalent)	Prevalence of MS (low *vs* . high) from published sources	Correlation between sausage preservation by smoking and high risk of MS
Sepcic et al 1993	Case-control	Croatia (*n* =46 people with MS; *n* =92 controls)	Food recall from birth until the first signs of the disease appeared	Risk of MS	Weekly consumption (3-5 times/week) of a traditional meal of potatoes with lard and smoked meat was associated with increased risk of MS
Zhang et al 2000	Cohort	Nurses' Health Study and Nurses Health study II (195 new cases of MS)	Food intakes from a food frequency questionnaire (g/day)	Risk of MS	No association between red meat or processed meat intake and risk of MS

P. Ghadirian, M. Jain, S. Ducic, B. Shatenstein, and R. Morisset, Nutritional factors in the aetiology of multiple sclerosis: a case-control study in Montreal, Canada. Int J Epidemiol. 27 (1998) 845-52.

E. Gusev, A. Boiko, K. Lauer, T. Riise, and T. Deomina, Environmental risk factors in MS: a case-control study in Moscow. Acta Neurol Scand. 94 (1996) 386-94.

K. Lauer, The risk of multiple sclerosis in the U.S.A. in relation to sociogeographic features: a factor-analytic study. J Clin Epidemiol. 47 (1994) 43-8.

K. Lauer, Sausage preservation methods and the prevalence of multiple sclerosis: An ecological study. Ecology of Food and Nutrition 2007 (2007) 1-11.

J. Sepcic, E. Mesaros, E. Materljan, and D. Sepic-Grahovac, Nutritional factors and multiple sclerosis in Gorski Kotar, Croatia. Neuroepidemiology 12 (1993) 234-40.

S.M. Zhang, W.C. Willett, M.A. Hernan, M.J. Olek, and A. Ascherio, Dietary fat in relation to risk of multiple sclerosis among two large cohorts of women. Am J Epidemiol. 152 (2000) 1056-64.
